# Pacific Coast Dental Congress

**Published:** 1896-01

**Authors:** 


### NOTICES.

### Pacific Coast Dental Congress.

SAN FRANCISCO, CALIFORNIA, Nov. 11th, 1895.

At a meeting of the General Committee of the Pacific Coast Dental Congress, held at San Francisco on October 30, 1895, it was resolved: "That the Congress meet in August, 1897, San Francisco to be the place of meeting, and that the American and Southern Dental Associations be invited to meet in San Francisco at that time and hold a joint or separate session, as may seem best."

C. L. GODDARD, D. D. S., Chairman.
H. G. RICHARDS, D. D. S., Secretary.
W. A. KNOWLS, M. D., D. D. S., Treasurer.
*General Committee.*

